# Tracking Risk Factors Related to an Outbreak of COVID-19 Among Healthcare Workers in a General Medicine Ward

**DOI:** 10.7759/cureus.48429

**Published:** 2023-11-07

**Authors:** Niranjana Nair, Ben Thomas Varghese, Hemica Hasan, Nagham Toba, Ghadah Alsharif, Poonam Panicker, Handan Celiloglu, Maida Balila, Ajaz Fakhri, Emily Lua, Amar H Khamis, Samuel B Ho

**Affiliations:** 1 Department of Medicine, Infection Control, and Quality Improvement, Mediclinic City Hospital, Dubai, ARE; 2 Department of Internal Medicine, College of Medicine, Mohammed Bin Rashid University of Medicine and Health Sciences, Dubai, ARE; 3 Department of Emergency Medicine, College of Medicine, Mohammed Bin Rashid University of Medicine and Health Sciences, Dubai, ARE; 4 Department of Pediatrics, College of Medicine, Mohammed Bin Rashid University of Medicine and Health Sciences, Dubai, ARE; 5 Department of Epidemiology and Public Health, College of Medicine, Mohammed Bin Rashid University of Medicine and Health Sciences, Dubai, ARE

**Keywords:** hospital-acquired infection, infection control, healthcare worker, sars-cov-2, covid-19

## Abstract

Background

An outbreak of severe acute respiratory syndrome coronavirus 2 (SARS-CoV-2) infection occurred in a medical ward involving patients and hospital staff from May to June 2020.

Aim

The aim of this study is to determine risk factors related to the outbreak of SARS-CoV-2 in six healthcare workers (HCWs) in a medical ward with initially unrecognized coronavirus disease 2019 (COVID-19) positive patients.

Methods

A retrospective cross-sectional study was conducted using a comprehensive questionnaire and personal interviews to determine the risk factors for COVID-19 infection in HCWs.

Findings

A total of 6/34 HCWs were diagnosed with COVID-19 in a medical ward. There were no differences between COVID-19 negative HCWs and COVID-19 positive HCWs in terms of mean duration of hours worked in the unit during the cluster event (180.2 vs 177.5 hours) (p>0.05), mean total time spent in contact with COVID-19 positive patients (12.8 vs 10.5 hours) (p>0.05), mean total time spent on aerosol-generating procedures (1.9 vs 0.9 hours) (p>0.05), and mean total time spent on non-aerosol generating procedures (10.9 vs 9.6 hours ) (p>0.05). There was no difference in exposure to COVID-19 positive family members among the HCWs (33% vs 3.7%, p=0.08). In contrast, exposure to COVID-19 positive contacts in the community was significantly greater in infected vs non-infected HCWs (16.7% vs 0%, p=0.03).

Conclusion

There was no significant difference in risk factors for contracting SARs-CoV2 among HCWs due to hospital exposures. COVID-19 positive HCWs were more likely to be exposed to positive individuals in their households and community, indicating that the source of SARS-CoV-2 infection came from outside the hospital.

## Introduction

The first human case of severe acute respiratory syndrome coronavirus 2 (SARS-COV-2) was reported in December 2019 in Wuhan, China, and it was subsequently declared as a global pandemic by the World Health Organization (WHO) in March 2020. Since then, SARS-COV-2 has been contracted by more than 100 million population worldwide and has caused more than 2 million deaths [1]. High infection rates among healthcare workers (HCWs) were initially reported in China, and concern about HCWs became a worldwide issue [2]. In March 2020, WHO released guidelines on infection prevention and control in healthcare facilities [3]. The first national guidelines on the clinical management and treatment of coronavirus disease 2019 (COVID-19) were released in the UAE in March 2020, and updated in April 2020, when the total cumulative cases in the UAE reached 1,264. These included guidelines for infection control measures for suspected or confirmed COVID-19 cases in healthcare facilities, specifically including precautions related to aerosol-generating procedures (AGPs), contact precautions, patient transport and isolation, and personal protective equipment (PPE) use [4]. The effectiveness of these infection control measures has not been evaluated, and, to date, a few studies have examined quality improvement measures in healthcare facilities related to preventative measures pertaining to COVID-19 during this early phase of the pandemic.

Our study investigates a SARS-CoV-2 outbreak that occurred in a private medical ward involving patients and hospital staff from May to June 2020. This study aimed to shed light on the possible risks of transmission of SARS-CoV-2 infection in healthcare and other settings. This article was previously presented as a meeting abstract at the 4th Annual Mediclinic Middle East Research Conference on May 27, 2021 [5].

## Materials and methods

**Figure 1:**
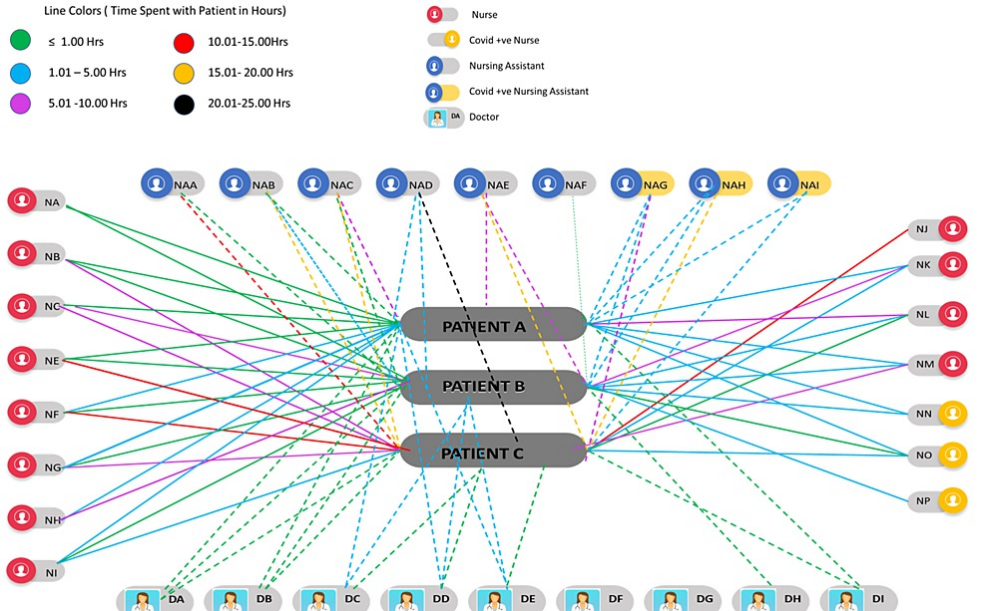
Contact tracing between COVID-19 positive patients and healthcare workers during a cluster event in the medical ward

This is a retrospective cross-sectional study that identified the risk factors for acquiring COVID-19 in HCWs by comparing exposure between COVID-19 positive and negative HCWs in a cluster event at a multidisciplinary private hospital. The cluster event took place in the medical ward where HCWs inadvertently encountered COVID-19 positive patients, as illustrated in Figure 1. The medical ward was a 31-bed unit that was designated as a non-COVID-19 ward. Any patient that tested positive for COVID-19 was transferred to a specialized ward. There were 34 staff members working on the unit during April to May 2020. The staff comprised six nurses, nine certified nursing assistants, and nine hospitalists. The study was approved by the Dubai Healthcare City Authority - Regulatory (DHCR) Research Ethics Committee (DHCR-REC-2021-36 MCH-GMW) and the Mediclinic Middle East (MCME) Research & Ethics Committee (MCME.CR.177.MCIT.2020).

Study timeline

**Figure 2:**
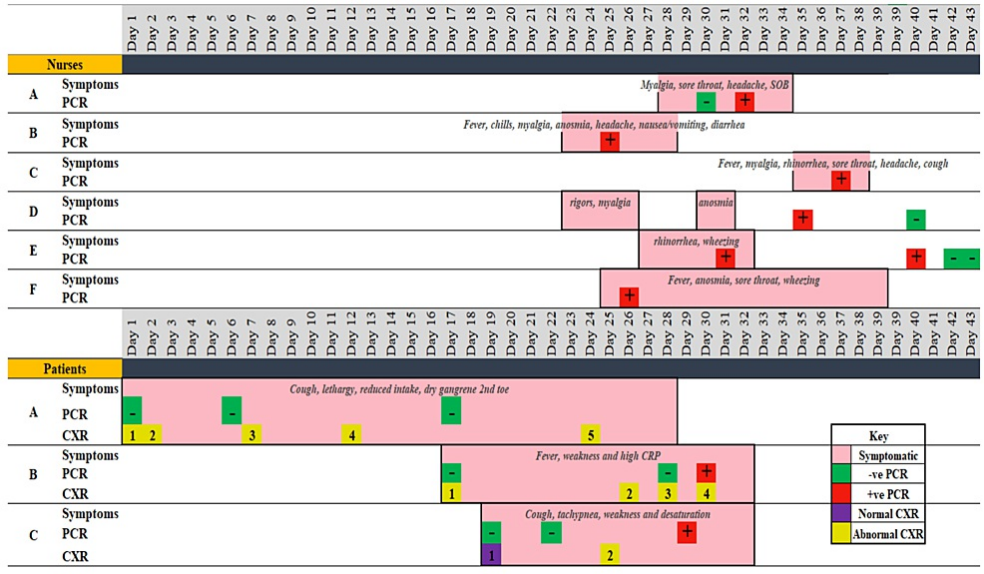
Healthcare workers and patients' timeline of events PCR, polymerase chain reaction; CXR, chest X-ray

To analyze the progression and spread of the COVID-19 infection outbreak within this unit, a timeline of events was mapped (Figure 2). A total of six HCWs (HCW-A, HCW-B, HCW-C, HCW-D, HCW-E, HCW-F) tested positive in the unit, all preceding the patients testing COVID-19 positive on polymerase chain reaction (PCR). However, all patients were clinically symptomatic at the time of admission, prior to the HCWs testing COVID-19 positive on PCR. HCW-A, HCW-B, and HCW-C were nurses in the ward, and HCW-D, HCW-E, and HCW-F were nursing assistants. A total of three patients (patient A, patient B, and patient C) eventually tested positive on the ward. Questions to be answered by this investigation included the following: Were the patients the source of this outbreak within the ward? Did the HCWs acquire the infection outside of the hospital or from other workers in the ward?

Case descriptions

Patient A is an 87-year-old male with known ischemic heart disease, diabetes, and past cerebrovascular accident, who was admitted to the ward on April 25 with cough, lethargy, reduced fluid intake, and urinary tract infection. His chest X-ray (CXR) on admission showed right upper lobe infiltrations, and right lower lobe bronchiectasis and infiltrates; subsequently, he was diagnosed with right-sided pneumonia. His SARS-CoV-2 PCR was negative on April 25, and his second and third SARS-CoV-2 PCR tests were also negative on April 30 and May 11, respectively; however, he was detected to have COVID-19 on May 30 with a positive PCR test.

Patient B was admitted to the ward with methicillin-resistant Staphylococcus aureus sepsis and had symptoms of fever and weakness with a high C-reactive protein. His CXR revealed right upper lobe reticular nodular opacifications with no other findings, and the SARS-CoV-2 PCR was negative on May 11 and May 22. On May 24, his CXR revealed bilateral mild and lower zone infiltrations with right pleural effusion, and his SARS-CoV-2 PCR was positive. Due to his unstable condition, he had to be intubated and put on a ventilator.

Patient C is an 85-year-old male with a previous history of hypertension, cerebrovascular accident, and dementia, who was admitted on May 13 with cough, tachypnea, lethargy, and an oxygen saturation of 83%. His management included piperacillin-tazobactam, azithromycin, and frequent nebulization. His CXR on admission was clear, and a repeat CXR on May 19 showed left lower lobe infiltrates. SARS-CoV-2 PCR on admission was not detected, and he remained negative on repeat test three days later; he was found to be SARS-CoV-2 PCR positive on May 23. His symptoms deteriorated prompting the requirement of intubation on May 22. He was transferred to a different hospital four days after his intubation.

Patient’s timeline of events

Patient A was admitted to the ward on day 1 and was tested for COVID-19 a total of three times. His tests were negative during his admission to the hospital for two weeks, where he underwent a total of three COVID-19 PCR tests (days 1, 6, and 17). During the same period, he underwent four CXRs, which showed signs of progressing lung infection that transformed into pneumonia on day 7 and remained unchanged until day 24. The patient was found to be positive on day 36, that is, 11 days after the first HCW (HCW-B) was detected to be positive (day 25). He was then transferred to another facility for further care.

Patient B was admitted on day 17 and was tested three times. His first two COVID-19 PCR tests were negative on days 17 and 29. His CXR meanwhile showed signs of lung infection (opacifications and pleural effusions). His first positive PCR was on day 30, that is, five days after the first positive PCR of our HCW (HCW-B day 25). The patient was transferred on day 32 to a different facility for further care. The patient recovered and had a negative PCR on day 48.

Patient C was admitted on day 19 and underwent three COVID-19 PCR tests. The first two tests were negative (days 19 and 22). He turned positive on day 29, that is, four days after the first positive PCR of our HCW (HCW-B day 25). His first CXR on admission was normal, but a repeat CXR on day 25 showed left lower lobe infiltrates. He was then transferred to another facility for further care.

Healthcare workers’ timeline of events

All HCWs underwent routine COVID-19 screening and were additionally tested if they had any symptoms. They all filled out a questionnaire to identify in-hospital and community exposure (see the Appendix for the questionnaires). In this case, all the nurses tested positive after experiencing symptoms. HCW-B was the first HCW to have a positive PCR result on day 25. HCW-F then tested positive the following day (day 26). HCW-E and HCW-A tested positive one day apart (days 31 and 32, respectively). Finally, HCW-D and HCW-C tested positive on days 35 and 37. All the HCWs and patients tested positive in the span of 10 days.

Nurses’ symptomatology

All six HCWs (HW-A, HW-B, HW-C, HW-D, HW-E, and HW-F) tested positive for SARS-CoV-2 on PCR. In all cases, symptom onset preceded PCR positivity by 1 to 10 days.

HW-B and HW-D were the first to develop symptoms among the cohort. HW-A (nurse N) developed symptoms on May 22, which lasted for seven days. Her symptoms consisted of myalgia, sore throat, headache, and shortness of breath.

HW-B (nurse O) developed symptoms on May 17, such as fever, chills, myalgia, anosmia, headache with nausea, vomiting, and diarrhea. Her symptoms persisted for seven days.

HW-C (nurse P) reported symptoms on May 29, which lasted for less than a week. Her symptoms were fever, myalgia, rhinorrhea, sore throat, headache, and cough.

HW-D (nursing assistant G) showed symptoms of rigors and myalgia on May 17. She then developed anosmia eight days later.

HW-E (nursing assistant H) developed symptoms of rhinorrhea and wheezing on May 21, which lasted for six days.

Exposure duration

HW-B, HW-D, and HW-E were exposed to all three patients in the unit. HW-A was only exposed to patient A and patient B. HW-F was only exposed to patient B and patient C. HW-C was only exposed to patient B.

Outcome measures

In this study, we looked at the difference in exposure duration, personal protective equipment (PPE) use, and community exposures between the COVID-19 positive HCWs (cases) and COVID-19 negative HCWs (controls). We measured the total time spent in the COVID-19 ward and the total time spent in contact with the COVID-19 positive patients. The time spent with the COVID-19 positive patients was further categorized as total time spent conducting AGPs and total time spent conducting non-aerosol generating procedures (NAGPs).

The PPE use between the cases and controls when in contact with each patient was measured by comparing the use of masks (single surgical mask, multiple surgical masks, and N95 mask), aprons, gowns, gloves, face shields, shoe covers, and hand washing before and after patient contact.

Community risk of acquiring COVID-19 was compared between cases and controls by retrospectively looking at the presence or absence of risk factors during the time of the cluster event, such as traveling, large group gatherings, visiting hotels, restaurants, or swimming pools. Community risk was also measured by comparing the housing conditions between the HCWs by assessing the number of adults and children they lived with. Direct community exposure was defined as contact with a COVID-19 positive patient in the community or at home.

All these variables were compiled into a single questionnaire, which was distributed to the nurses and answered retrospectively based on their memory of how much time they spent on each of the variables.

Analysis

Categorical data were presented as frequencies and proportions, and continuous data were presented as means and standard deviations. Baseline characteristics of HCWs who were infected and those not infected were compared using the Mann-Whitney test for continuous data. Meanwhile, exact Fischer’s test was used for categorical variables. Average ratios and prevalence ratios between infected and non-infected, as well as their corresponding confidence intervals, were calculated for all the variables in the study when applicable. A p-value of less than 0.05 was considered statistically significant in all the tests. Analysis was performed using Statistical Package for the Social Sciences (SPSS) for Windows Version 25 (IBM Corp., Armonk, NY).

## Results

Demographics

A total of 34 HCWs were screened in the unit while identifying the cluster event: 16 were nurses, nine were physician hospitalists, and nine were nursing assistants. All HCWs had contact with at least one of the three COVID-19 positive patients (patient A, patient B, and patient C). Of the HCWs, 82% were female, and 74% were under the age of 40 years. A total of six out of 34 HCWs were positive for SARS-CoV-2 on PCR: 3/16 nurses, 0/9 doctors, and 3/9 nursing assistants. There was no statistically significant difference in terms of risk of acquiring COVID-19 between HCWs who were >40 years and <40 years of age (p>0.05) or in terms of gender of the HCWs (p>0.05).

Exposures

The measured exposures included the difference in exposure duration, PPE use, and community exposures between the COVID-19 positive HCWs (cases) and COVID-19 negative HCWs (controls).

There was no difference between COVID-19 positive HCWs (cases) and COVID-19 negative HCWs (controls) in terms of mean duration of hours worked that month in the unit during the cluster event (177.50 [SD: 28.42] hours vs 176.89 [SD: 30.73] hours; average ratio (AR): 1.00; p=0.56) or mean total time spent in contact with the 3 patients (10.53 [SD: 9.74] hours vs 9.15 [SD: 9.00] hours; AR: 1.15; p=0.54). There was no difference in mean total time spent on AGPs for three patients (0.95 [SD: 0.94] hours vs 1.45 [SD: 1.39] hours; AR: 0.65; p=0.51) and mean total time spent on NAGPs for three patients (9.58 [SD: 10.06] hours vs 7.70 [SD: 8.87] hours; AR: 1.24; p=0.46). When comparing the different types of AGPs and NAGPs, there was no difference between the cases and controls (Table 1). There was also no difference in PPE use between cases and controls when in contact with any of the patients. None of the HCWs wore multiple surgical masks, and all HCWs washed their hands before and after patient contact.

**Table 1:** Total time spent on AGP and NAGP by healthcare workers around COVID-19 positive patients. AGP, aerosol-generating procedures; NAGP, non-aerosol-generating procedures; SD, standard deviation

Procedures (hours per month)	Cases, n	Cases, mean (SD)	Controls, n	Controls, mean (SD)	Ratio of cases:controls	p-Value
Total time in ward	6	177.50 (28.42)	27	176.89 (30.73)	1.00	0.56
AGP						
Oxygen	6	0.46 (0.36)	25	0.43 (0.45)	1.06	0.688
Nebulization	6	0.28 (0.38)	25	0.54 (0.63)	0.56	0.522
Suction	6	0.15 (0.27)	25	0.42 (0.65)	0.37	0.504
High-flow nasal oxygen	6	0.06 (0.13)	25	0.14 (0.43)	0.39	0.885
NAGP						
Nasogastric tube insertion	6	0.00 (0.00)	25	0.02 (0.10)	0.00	0.624
History and evaluation	6	0.00 (0.00)	25	0.41 (0.77)	0.00	0.092
Canulation	6	0.01 (0.03)	25	0.17 (0.31)	0.08	0.154
Blood/air blood gas	6	0.10 (0.13)	25	0.32 (0.44)	0.30	0.200
Nasal swab	6	0.03 (0.04)	25	0.01 (0.04)	2.09	0.240
Feeding	6	0.13 (0.31)	25	0.39 (0.72)	0.32	0.454
Medication	6	0.79 (1.23)	25	1.04 (1.64)	0.76	0.717
Re-positioning	6	6.53 (6.64)	25	4.31 (6.23)	1.51	0.209
Bathing	6	2.08 (5.10)	25	0.79 (2.39)	2.63	0.668
Dressing	6	0.08 (0.20)	24	0.40 (0.85)	0.21	0.370
Vital signs	6	0.00 (0.00)	25	0.35 (1.11)	0.00	0.381
Total AGP + NAGP	6	10.53 (9.74)	27	9.15 (9.00)	1.15	0.544

Individual patient contact time and PPE use

When looking at exposure to each patient separately, there was no difference in mean total time spent in contact with patient A (1.93 [SD: 1.85] hours vs 1.71 [SD: 2.02] hours; AR: 1.13; p=0.78), mean total time spent on AGPs for patient A (0.305 [SD: 0.48] hours vs 0.52 [SD: 0.75] hours; AR: 0.59; p=0.50), and mean total time spent on NAGPs for patient A (1.63 [SD: 1.57] hours vs 1.18 [SD: 1.90] hours; AR: 1.37; p=0.48). There was also no difference in specific AGPs and NAGPs between cases and controls when in contact with patient A (Table 2). There was also no difference in PPE use between cases and controls when in contact with patient A (Table 3).

**Table 2:** Hours of contact with patient A. AGP, aerosol-generating procedures; NAGP, non-aerosol-generating procedures; SD, standard deviation

Procedures	Cases, n	Cases, mean (SD)	Controls, n	Controls, mean (SD)	Ratio of cases:controls	p-Value
AGP						
Oxygen	4	0.19 (0.22)	21	0.16 (0.20)	1.19	0.620
Nebulization	4	0.17 (0.24)	21	0.18 (0.30)	0.92	0.969
Suction	4	0.11 (0.13)	21	0.22 (0.48)	0.48	0.834
High-flow nasal oxygen	4	0.00 (0.00)	21	0.11 (0.32)	0.00	0.288
NAGP						
Nasogastric tube insertion	4	0.00 (0.00)	21	0.02 (0.11)	0.00	0.663
History and evaluation	4	0.00 (0.00)	21	0.15 (0.24)	0.00	0.190
Canulation	4	0.00 (0.00)	21	0.02 (0.04)	0.00	0.431
Blood/air blood gas	4	0.02 (0.04)	21	0.08 (0.17)	0.24	0.622
Nasal swab	4	0.02 (0.04)	21	0.01 (0.02)	2.63	0.392
Feeding	4	0.00 (0.00)	21	0.00 (0.00)	-	1.000
Medication	4	0.5 (0.71)	21	0.12 (0.24)	4.20	0.300
Re-positioning	4	1.52 (1.03)	21	0.92 (1.62)	1.66	0.080
Bathing	4	0.63 (0.68)	21	0.20 (0.53)	3.09	0.486
Dressing	4	0.00 (0.00)	21	0.00 (0.00)	-	1.000
Vital signs	4	0.00 (0.00)	21	0.03 (0.90)	0.00	0.529
Total AGP + NAGP	6	1.93 (1.85)	27	1.71 (2.02)	1.13	0.778

**Table 3:** PPE use by healthcare workers when in contact with patient A. PPE, personal protective equipment

PPE used	Cases, n	Cases	Controls, n	Controls	Prevalence ratio	p-Value
Single surgical mask	4	3/4 (75%)	21	8/21 (38.1%)	1.96	0.21
Multiple surgical masks	4	0	21	0	0.00	-
N95 mask	4	1/4 (25%)	21	17/21 (81%)	0.31	0.053
Apron	4	0/4 (0%)	21	1/21 (4.8%)	0.00	0.84
Gown	4	4/4 (100%)	21	16/21 (76.2%)	1.31	0.38
Gloves	4	4/4 (100%)	21	21/21 (100%)	1.00	-
Face shield	4	2/4 (50%)	21	11/21 (52.4%)	0.95	0.67
Shoe cover	4	3/4 (75%)	21	10/21 (47.6%)	1.58	0.33
Hand washing						
Before	4	4/4 (100%)	21	21/21 (100%)	1.00	-
After	4	4/4 (100%)	21	21/21 (100%)	1.00	-

There was no difference in mean total time spent in contact with patient B (2.24 [SD: 1.49] hours vs 1.68 [SD: 2.17] hours; AR: 1.33; p=0.154), mean total time spent on AGPs for patient B (0.36 [SD: 0.31] hours vs 0.33 [SD: 0.27] hours; AR: 1.11; p=0.52), and mean total time spent on NAGPs for patient B (1.88 [SD: 1.61] hours vs 1.36 [SD: 1.86] hours; AR: 1.38; p=0.11). There was also no difference in specific AGPs and NAGPs between cases and controls when in contact with patient B (Table 4). There was also no difference in PPE use between cases and controls when in contact with patient B (Table 5).

**Table 4:** Hours of contact with patient B. AGP, aerosol-generating procedures; NAGP, non-aerosol-generating procedures; SD, standard deviation

Procedures	Cases, n	Cases, mean (SD)	Controls, n	Controls, mean (SD)	Ratio of cases:controls	p-Value
AGP						
Oxygen	6	0.24 (0.21)	22	0.16 (0.21)	1.52	0.384
Nebulization	6	0.07 (0.08)	22	0.10 (0.15)	0.68	0.902
Suction	6	0.00 (0.00)	22	0.08 (0.20)	0.00	0.348
High-flow nasal oxygen	6	0.06 (0.13)	22	0.05 (0.15)	1.03	0.890
NAGP						
Nasogastric tube insertion	6	0.00 (0.00)	17	0.00 (0.00)	-	1.000
History and evaluation	6	0.00 (0.00)	22	0.16 (0.32)	0.00	0.091
Canulation	6	0.00 (0.00)	22	0.12 (0.25)	0.00	0.092
Blood/air blood gas	6	0.08 (0.11)	22	0.08 (0.11)	1.05	0.803
Nasal swab	6	0.01 (0.03)	22	0.00 (0.00)	-	0.056
Feeding	6	0.00 (0.00)	22	0.00 (0.00)	-	1.000
Medication	6	0.29 (0.46)	22	0.23 (0.45)	1.26	0.779
Re-positioning	6	1.15 (1.21)	22	0.85 (1.37)	1.35	0.369
Bathing	6	0.33 (0.82)	22	0.14 (0.54)	2.44	0.602
Dressing	6	0.00 (0.00)	16	0.00 (0.00)	-	1.000
Vital signs	6	0.00 (0.00)	22	0.09 (0.30)	0.00	0.348
Total AGP + NAGP	6	2.24 (1.49)	27	1.68 (2.17)	1.33	0.154

**Table 5:** PPE use by healthcare workers when in contact with patient B PPE, personal protective equipment

PPE used	Cases, n	Cases	Controls, n	Controls	Prevalence ratio	p-Value
Single surgical mask	6	5/6 (83.3)	24	18/24 (75%)	1.11	0.57
Multiple surgical masks	6	0	24	0	0.00	-
N95 mask	6	1/6 (16.7%)	24	7/24 (29.2%)	0.57	0.48
Apron	6	0/6 (0%)	24	2/24 (8.3%)	0.00	0.63
Gown	6	4/6 (66.7%)	24	10/24 (41.7%)	1.60	0.26
Gloves	6	6/6 (100%)	24	21/24 (87.5%)	1.14	0.50
Face shield	6	1/6 (16.7%)	24	3/24 (12.5%)	1.34	0.61
Shoe cover	6	2/6 (33.3%)	24	5/24 (20.8%)	1.60	0.43
Hand washing						
Before	6	6/6 (100%)	24	22/24 (91.7%)	1.09	0.63
After	6	6/6 (100%)	24	22/24 (91.7%)	1.09	0.63

There was no difference in mean total time spent in contact with patient C (6.36 [SD: 7.64] hours vs 5.76 [SD: 6.95] hours; AR: 1.10; p=0.981), mean total time spent on AGPs for patient C (0.28 [SD: 0.60] hours vs 0.60 [SD: 0.92] hours; AR: 0.46; p=0.50), and mean total time spent on NAGPs for patient C (6.08 [SD: 7.73] hours vs 5.16 [SD: 6.63] hours; AR: 1.18; p=0.90). There was also no difference in specific AGPs and NAGPs between cases and controls when in contact with patient C (Table 6). There was also no difference in PPE use between cases and controls when in contact with patient C (Table 7).

**Table 6:** Hours of contact with patient C. AGP, aerosol-generating procedures; NAGP, non-aerosol-generating procedures; SD, standard deviation

Procedures	Cases ,n	Cases, mean (SD)	Controls, n	Controls, mean (SD)	Ratio of cases:controls	p-Value
AGP						
Oxygen	4	0.15 (0.24)	22	0.19 (0.24)	0.78	0.849
Nebulization	4	0.15 (0.24)	22	0.34 (0.53)	0.43	0.727
Suction	4	0.13 (0.25)	22	0.19 (0.38)	0.66	0.891
High-flow nasal oxygen	4	0.00 (0.00)	22	0.00 (0.00)	-	1.000
NAGP						
Nasogastric tube insertion	4	0.00 (0.00)	16	0.00 (0.00)	-	1.000
History and evaluation	4	0.00 (0.00)	22	0.16 (0.30)	0.00	0.248
Cannulation	4	0.02 (0.04)	21	0.07 (0.13)	0.30	0.743
Blood/air blood gas	4	0.00 (0.00)	22	0.20 (0.39)	0.00	0.104
Nasal swab	4	0.00 (0.00)	22	0.01 (0.02)	0.00	0.538
Feeding	4	0.19 (0.38)	21	0.47 (0.76)	0.40	0.605
Medication	4	0.25 (0.50)	22	0.84 (1.62)	0.30	0.477
Re-positioning	4	6.54 (5.23)	22	3.17 (4.62)	2.06	0.075
Bathing	4	2.00 (4.00)	22	0.57 (1.90)	3.49	0.534
Dressing	4	0.13 (0.25)	22	0.43 (0.88)	0.29	0.598
Vital signs	3	0.00 (0.00)	22	0.28 (0.85)	0.00	0.505
Total AGP + NAGP	6	6.36 (7.64)	27	5.76 (6.95)	1.10	0.981

**Table 7:** PPE use by healthcare workers when in contact with patient C. PPE, personal protective equipment

PPE used	Cases, n	Cases	Controls, n	Controls	Prevalence ratio	p-Value
Single surgical mask	6	4/6 (66.7%)	25	16/25 (64%)	1.04	0.65
Multiple surgical masks	6	2/6 (33.3%)	25	3/25 (12%)	2.78	0.24
N95 mask	6	1/6 (16.7%)	25	8/25 (32%)	0.52	0.42
Apron	6	1/6 (16.7%)	25	1/25 (4%)	4.18	0.36
Gown	6	4/6 (66.7%)	25	13/25 (52%)	1.28	0.43
Gloves	6	6/6 (100%)	25	22/25 (88%)	1.14	0.51
Face shield	6	1/6 (16.7%)	25	8/25 (32%)	0.52	0.42
Shoe cover	6	2/6 (33.3%)	25	5/25 (20%)	1.65	0.41
Hand washing						
Before	6	6/6 (100%)	25	25/25 (100%)	1.00	-
After	6	6/6 (100%)	25	25/25 (100%)	1.00	-

The community exposures between cases and controls are tabulated in Table 8 and Table 9. Interestingly, the only significant exposure that the COVID-19 positive HCWs had that was greater than the COVID-19 negative HCWs was in the community exposure, with the prevalence of contact with a COVID-19 positive individual in the community being 16.7% in the cases versus 0% in the controls (p=0.03). Moreover, there was a tendency toward a greater prevalence of the cases having a COVID-19 positive spouse compared to controls (33% vs 3.7%; p=0.08). There was no difference between cases and controls in the prevalence of other community exposures such as staying at a hotel (0% vs 7.4%; p=0.50), going out to restaurants (0% vs 7.4%; p=0.50), or going out swimming (0% vs 3.4%; p=0.64). None of the HCWs traveled or had a gathering of more than 10 people, and all the HCWs went out shopping; therefore, these risk factors could not be compared. There was also no statistically significant difference between cases and controls in their housing conditions, with the mean number being similar in terms of adults they lived with (3.83 [SD: 2.56] vs 3.37 [SD: 2.02]; p=0.77), children they lived with (0.33 [SD: 0.52] vs 0.89 [SD: 1.28]; p=0.43), number of people sharing a kitchen (1 [SD: 0] vs 0.93 [SD: 0.27]; p=0.50), and number of people sharing a bathroom (0.67 [SD: 0.52] vs 0.63 [SD: 0.49]; p=0.87).

**Table 8:** Possible out-of-hospital exposure in healthcare workers

Community exposure	Cases/control, n	Cases	Controls	Prevalence ratio	p-Value
Recent travel	6/27	0	0	0.00	1.00
Gathering with more than 10 people	6/27	0	0	0.00	1.00
Recent shopping/grocery shopping	6/27	6/6 (100%)	27/27 (100%)	1.00	1.00
Hotel stay	6/27	0	2/27 (7.4%)		0.50
Restaurant visit	6/27	0/6 (0%)	2/27 (7.4%)	0.00	0.50
Exposure to a public swimming pool	6/27	0/6 (0%)	1/27 (3.4%)	0.00	0.64
Exposure to COVID-19 positive individuals in the community	6/27	1/6 (16.7%)	0/27 (0.0%)	0.00	0.03
Spouse with COVID-19	6/27	2/6 (33%)	1/27 (3.7%)	8.91	0.08

**Table 9:** Living condition of healthcare workers.

Living condition	Cases/controls, n	Cases, mean (SD)	Controls, mean (SD)	Average ratio	p-Value
No. of adults in the house	6/27	3.83 (2.56)	3.37 (2.02)	1.14	0.77
No. of children	6/27	0.33(0.52)	0.89 (1.28)	0.37	0.43
No. of people sharing a kitchen	6/27	1.00 (0.00)	0.93 (0.27)	1.08	0.50
No. of people sharing a bathroom	6/27	0.67 (0.52)	0.63 (0.49)	1.06	0.87

## Discussion

In this cohort, 34 HCWs had contact with at least one COVID-19 positive patient; six of these 34 HCWs tested positive for SARS-CoV-2 on a nasal swab PCR. There was no statistical difference in contracting COVID-19 and the age of the HCWs, nor in their gender. There was no statistically significant difference in the exposure duration or PPE use and contracting COVID-19 among any of the three patients. Exposure assessment included time spent in the COVID-19 ward and total time spent in contact with the COVID-19 positive patients, with the latter including total time spent conducting AGP and total time spent conducting NAGP. There was no statistical significance in exposure and contracting COVID-19 from any of the patients.

However, exposure to a COVID-19 positive individual in the community was associated with a statistically significant higher prevalence of contracting COVID-19 in HCWs (p=0.03). There was a greater prevalence of COVID-19 positive spouses among the cases in comparison to the controls (33% vs 3.7%; p=0.08). Other community exposures, including staying at a hotel, going out to restaurants, or going out swimming, were not different between cases and controls. There was also no statistically significant difference between cases and controls in their housing conditions.

During the first phase of the pandemic, healthcare systems were overburdened by the rising COVID-19 cases. HCWs struggled with increased psychological stress, fatigue, and long working hours. Additionally, there was a global shortage of PPE available for HCWs [6], and they were exposed to suspected COVID-19 infected patients with minimal to no PPE. Therefore, during the initial stages of the pandemic, HCWs were at an increased risk of acquiring COVID-19 [7,8]. The present study was conducted in HCWs without any PPE shortage.

Lessons from the first phase of the pandemic taught HCWs the precautionary steps needed to prevent an outbreak, such as screening for SARS-CoV-2, isolating and quarantining COVID-19 positive patients, screening of HCWs who work with COVID-19 positive patients, maintaining social distancing [9-11], using PPE in wards housing COVID-19 positive patients, and continuous use of mask in the hospital [12,13]. The HCWs in the present study were equipped with all these lessons learnt from the initial stages of the pandemic.

The literature has shown that the risk of HCWs acquiring COVID-19 in the intensive care setting is low [14,15], possibly owing to the extensive PPE use and awareness of the patients’ COVID-19 infection status. However, there are growing concerns that the risk to HCWs may be arising from contact with patients at the early stages of the infection who are pre-symptomatic or asymptomatic [11].

Several studies in the literature that showed an increased risk of HCWs contracting the COVID-19 infection were conducted during the first wave of the pandemic, before the establishment of universal masking protocols, or in settings where access to face masks was limited [6,16,17]. In these studies, the in-hospital and community risk factors were not measured. A study by Wilkins et. al. on 6,510 HCWs in a well-resourced healthcare system showed that the risk of HCWs testing positive for COVID-19 was lower than previous estimates when community exposure was accounted for [18]. Another study that investigated the potential source of infection in HCW observed a trend where those with a larger household size tended to have more frequently detectable antibodies against SARS-CoV-2 [19]. A meta-analysis conducted by Gómez-Ochoa et al. evaluated the potential sources of COVID-19 infections among healthcare workers, assessing both nosocomial and community-acquired risk factors [20]. There seem to be no significant differences in infection rates of HCW groups working in high, intermediate, and low-exposure risk settings [21,22]. Additionally, the proportion of HCWs who tested positive for COVID-19 was the same in HCWs with patient-facing roles and non-patient-facing roles. The meta-analysis also revealed that community transmission has a relevant role in HCW infection rates [19,20,22,23]. The findings of our study are consistent with this meta-analysis. We encountered the consistent condition of increased community exposure being a significant risk factor for acquiring the infection. Our literature search revealed a substantial need for data on the risk factor disparities in HCW [24].

Finally, most of the published literature does not identify the specific workplace risk factors such as time spent working with COVID-19 positive patients. Our study used a novel systematic survey of potential hospital and community risk factors that was applied to both infected and non-infected HCWs. We believe that this survey could be of use to other hospitals investigating similar incidences of COVID-19 infections identified in HCWs (Appendix).

Strength and limitations

One of the strengths of this study was the extensiveness of the survey, which attempted to cover the majority of patient interaction variables. Also, there was a high completion rate of the survey, which limited any gap in information. A limitation is the small sample size of the cluster event, where only six of the HCWs could be included as cases; this reduces the power of the study, but it is still valuable in observing and analyzing the trends in this event. Since the survey was administered retrospectively to all HCWs involved, there is a possibility of recall bias in accurately documenting the information in the survey, but the administration was done soon after the cluster event, reducing the risk of bias.

It is possible that other important risk factors were not surveyed in the present study. Socialization between HCWs, such as for face-to-face meetings and meals, is a possible mechanism of transmission between HCWs [25]; however, this was not measured in the present study. This may have been a small factor because hospital policy prohibited these types of interactions. A previous study showed that socializing between HCWs and the lack of PPE was strongly correlated with the risk of HCWs acquiring COVID-19 from patients but conducting AGPs was not [26].

Our study can further ease the HCW's fear of acquiring COVID-19, from false COVID-19 negative patients admitted to the hospital, due to the lack of significant difference in risk from patient contact between cases and controls. Continued vigilance and monitoring of hospital infection control practices related to COVID-19 remain increasingly important with the continued spread of potentially more transmissible new variants [27].

## Conclusions

In conclusion, our paper reports a cluster event of COVID-19 infection in a hospital ward associated with initially unrecognized COVID-19 positive patients. No significant difference was found in risk factors for contracting SARS-CoV-2 among HCWs due to hospital exposures. COVID-19 positive HCWs were more likely to be exposed to positive individuals in their households and community, indicating that the source of SARS-CoV-2 infection likely came from outside the hospital. However, these data do not rule out a yet-to-be-identified mechanism of transmission independent of the duration and type of exposures measured in this study.

## Appendices

Questionnaires 

**Table 10:** Monthly patient contact questionnaire

Patient contact hours in the last four weeks =_____________________________				
	Please define the time spent in hours:			
	1^st^ week	2^nd^ week	3^rd^ week	4^th^ week
Outpatient				
Inpatient				
Intensive care				
Emergency department				
Home screening				
Physiotherapy				
Administration				
Medical records				
Radiology				
Screening test				
Other specify (…..................................................)				

**Table 11:** Post-exposure symptomatology questionnaire

Have you experienced any of the following symptoms in the last four weeks?					
Symptoms	Circle if Yes or No	If yes, please define symptom onset (✔):			
		1^st^ week	2^nd^ week	3^rd^ week	4^th^ week
Fever > 38°C	Yes/No				
Subjective fever	Yes/No				
Chills	Yes/No				
Rigors	Yes/No				
Muscle aches (myalgia)	Yes/No				
Runny nose (rhinorrhea)	Yes/No				
Sore throat	Yes/No				
Now olfactory and taste disorder	Yes/No				
Headache	Yes/No				
Fatigue	Yes/No				
Cough	Yes/No				
Wheezing	Yes/No				
Shortness of breath (dyspnea)	Yes/No				
Difficulty breathing	Yes/No				
Chest pain	Yes/No				
Nausea or vomiting	Yes/No				
Abdominal pain	Yes/No				
Diarrhea (>3 loose stools per 24-hour period)	Yes/No				

**Table 12:** Procedures performed questionnaire AGP, aerosol-generating procedures; NAGP, non-aerosol generating procedures; IV intravenous

Procedures on COVID-19 positive patient		
	Circle if Yes or No	If yes, please define the no. of procedures and total time spent (hours)
AGP		
Oxygen	Yes/No	
Nebulization	Yes/No	
Suction	Yes/No	
High-flow nasal cannula	Yes/No	
NAGP		
Nasogastric tube insertion	Yes/No	
History and evaluation	Yes/No	
IV cannulation	Yes/No	
Venipuncture/blood gas	Yes/No	
Feeding	Yes/No	
Medication administration	Yes/No	
Re-positioning	Yes/No	
Bathing	Yes/No	
Dressing	Yes/No	
Vital signs	Yes/No	
Other aerosol-generating procedures	Yes/No	
Others specify (……...............................................)		
Total time		

**Table 13:** Personal protective equipment use questionnaire PPE, personal protective equipment

PPE use while interacting with COVID-19 positive patient	
PPE use	Did you use the following PPE during interaction with the patient?
Mask	Yes/No
Single surgical	Yes/No
Multiple surgical	Yes/No
N95	Yes/No
Apron	Yes/No
Gown	Yes/No
Gloves	Yes/No
Face shield	Yes/No
Shoe cover	Yes/No
Hand washing	Yes/No
Before	Yes/No
After	Yes/No

**Table 14:** Community exposure to COVID-19 questionnaire for healthcare workers

Community exposure in the last four weeks		
Exposure	y / n	
		Travel	Yes/No	
Gathering with >10 people	Yes/No	
Shopping/grocery	Yes/No	
Hotel	Yes/No	
Restaurant	Yes/No	
Swimming	Yes/No	
Did you have contact with a known COVID-19 case (probable or confirmed)?	Yes/No	
If yes, then specify by circling below: 1. Household contact 2. Community-associated contact 3. Healthcare-associated contact		
What is the total number of people in your household?	_____ Adults ______ Children ______	
No. of people sharing kitchen and bathroom	Share kitchen ______ Share bathroom	
